# Middle ear adenomatous neuroendocrine tumors: suggestion for surgical strategy

**DOI:** 10.1016/j.bjorl.2020.05.011

**Published:** 2020-06-15

**Authors:** Bingbin Xie, Shaorong Zhang, Chunfu Dai, Yuehui Liu

**Affiliations:** a The Second Affiliated Hospital of Nanchang University, Department of Otolaryngology Head and Neck Surgery, Nanchang, China; b Jiangxi Biomedical Engineering Research Center for Auditory Research, Nanchang, China; c Fudan University, Eye and Ear, Nose, and Throat Hospital, Department of Otology and Skull Base Surgery, Shanghai, China

**Keywords:** Adenomas, Carcinoid tumor, Ear, Middle, Skull, Temporal bone

## Abstract

**Introduction:**

Middle ear adenomatous neuroendocrine tumors are extremely rare neoplasms with epithelial and neuroendocrine differentiation, accounting for fewer than 2% of all middle and inner ear tumors. Universal standard surgical procedures for different stages of these tumors remain elusive due to the limitation of the small number of case reports or investigations.

**Objective(s):**

This study intends to investigate proper surgical strategies for patients with middle ear adenomatous neuroendocrine tumors.

**Methods:**

Six patients with middle ear adenomatous neuroendocrine tumors who were treated at the Second Affiliated Hospital of Nanchang University (Nanchang, China) and the Eye, Ear, Nose, and Throat Hospital of Fudan University (Shanghai, China) respectively. Clinical characteristics and management strategies of patients were reviewed. The mean follow-up time was 63.7 months (range, 13–153 months). All the information was collected from medical records and prognosis postoperatively.

**Results:**

Three patients underwent canal wall-up tympanomastoidectomy, including one patient with recurrence who underwent a previous tympanotomy; the other three patients underwent lateral temporal bone resection All of these patients were followed up with no evidence of recurrence or metastasis. Patients underwent canal wall-up surgery treatment accompanied with hearing function preservation measurements during follow-up periods.

**Conclusions:**

Complete surgical resection provided good results for patients with middle ear adenomatous neuroendocrine tumors. The ossicular chain should be removed. Because of the propensity for local recurrence and invasiveness, as well as regional or distant metastasis of these tumors, it is necessary to schedule long-term follow-up and an observation plan postoperatively.

## Introduction

Adenomas and carcinoid tumors of the middle ear are extremely rare neoplasms with epithelial and neuroendocrine differentiation, which are account for fewer than 2% of all middle and inner ear tumors. These tumors are still in much debate for their similarities, differences, etiologies, and prognoses, since they were first described in 1976 by Hyams and Michaels^1^ and further detailed in 1980 by Murphy and colleagues.^2^ Because the tumor presented both neuroendocrine and glandular histological features, numerous terms have been reported in the literature to describe this group of lesions, such as middle ear carcinoid tumor, adenomatous tumor of the middle ear, adeno-carcinoid tumor and middle ear adenomatous neuroendocrine tumors (MEANTs).^3^, ^4^, ^5^, ^6^, ^7^, ^8^

To date, approximately 160 cases of MEANTs have been reported in the English literature.^4^, ^8^ However, a large comprehensive evaluation of these tumors is still necessary in future investigation, in order to demonstrate the histomorphology, immunohistochemical reactivity, clinical behavior, treatment strategies and outcomes. Mostly, MEANTs shows indolent biological behavior, with a benign histologic appearance and slow local growth, and the primary recommended treatment is surgical excision in the majority of localized cases. However, more aggressive histologic patterns may also sometimes be observed, and these tumors can recur, metastasize to the lymph nodes and distant sites, and cause death.^4^ Similar to the variety of terminology of these lesions, the surgical strategies also have tremendous differences. Universal standard surgical procedures for different stages of these tumors remain elusive due to the limitation of the small number of case reports or investigations.

Herein, we present our experience on MEANTs, a review of the literature and a discussion of the management of patients with these tumors, in order to look for proper surgical strategies for MEANTs.

## Methods

After obtaining institutional review board and Ethics Committee approval (n° 2016[007]) from the Second Affiliated Hospital of Nanchang University (Nanchang, China) and the Eye, Ear, Nose, and Throat Hospital of Fudan University (Shanghai, China), a retrospective analysis was undertaken of all patients with MEANTs who were treated at both hospitals between April 2006 and December 2017. All information based on patients’ medical records and follow-up interviews, including clinical symptoms, extent of the lesion, previous treatments, surgical procedures and findings, histopathologic diagnosis, follow-up interval, recurrence information, and survival, was collected up to December 2019.

## Results

### Patient characteristics and clinical manifestations

In the present study, 6 patients were identified with MEANTs. Two patients were diagnosed as MEANTs primarily in our department, and 3 patients were diagnosed as secondarily in our department, after receiving initial treatment in local hospital. One patient was misdiagnosed as Chronic Suppurative Otitis Media (CSOM) preoperatively. Patient characteristics and clinical manifestations are presented in detail in Table 1.

**Table 1:** Characteristics and clinical manifestations of the 6 patients with MEANTs.

Features	Median/Average
*Age at diagnosis (median)*	43.5 (range from 30 to 62)

*Gender*	
Male	3
Female	3

*AC pure tone average (0.5, 1, 2, 4* *kHz) in dB*	47.7
*BC pure tone average (0.5, 1, 2, 4* *kHz) in dB*	23.3

Symptoms	N° of patients	% of patients
*Hearing loss*		
Gradual hearing loss	6	100
Sudden hearing loss	0	0

*Aural fullness*	5	83.3
*Tinnitus*	4	66.7
*Otorrhea*	2	33.3
*Otalgia*	0	0
*Bloody discharge*	0	0
*Carcinoid syndrome*	0	0
*Facial weakness or paralysis*	0	0
*Vertigo*	0	0

MEANTs, Middle Ear Adenomatous Neuroendocrine Tumors.

### Preoperative evaluation and treatment strategies

Case 1 presented with a small perforation in the pars tensa near the posteroinferior quadrant, with granulation tissue in the tympanum and sticky purulent discharge. This patient was misdiagnosed as CSOM preoperatively, underwent a CWU tympanomastoidectomy and ossicular chain reconstruction with Partial Ossicular Replacement Prosthesis (PORP) (Table 2). The histopathology confirmed a diagnosis of MEANT postoperatively.

**Table 2:** Preoperative evaluation and treatment strategies for patients.

Case	Sex	Age	Clinical manifestation	Disease location	TNMS	Treatments	Margins	Follow-up (M)	Outcome
1	M	43	HL, AF, T, Otorrhea	ME	T1N0M0S0^a^	CWU TM	None^c^	148	NED
2	F	44	T	ME	T1N0M0S0	CWU TM	Negative	49	NED
3^b^	F	30	T, AF	ME	T1N0M0S0^a^	Tympanotomy & lesion excision	Negative	81	Recurrent
4	M	40	HL, AF	EAC	T2N0M0S0	LTBR & superficial parotidectomy	Negative	45	NED
5	M	62	HL, AF, T, Otorrhea	ME & EAC	T2N0M0S0	LTBR	Negative	21	NED
6	M	44	HL, AF	ME & EAC	T2N0M0S0	LTBR	Negative	8	NED

a Postoperative stage.

b Case 3 with local recurrence 38 months postoperative, and underwent second surgery, CWU mastoidectomy and type I tympanoplasty with an intact ossicles. 02/10/2015 recurrent, Reddish lesion EAC CWU + Tympanoplasty.

c Case 1 was misdiagnosed patients without margins report. HL, Hearing Loss; AF, Aural Fullness; T, Tinnitus; ME, Middle Ear; EAC, External Auditory Canal; CWU, Canal Wall-Up; TM, Tympanomastoidectomy; LTBR, Lateral Temporal Bone Resection; NED, No Evidence of Disease.

**Figure 1:**
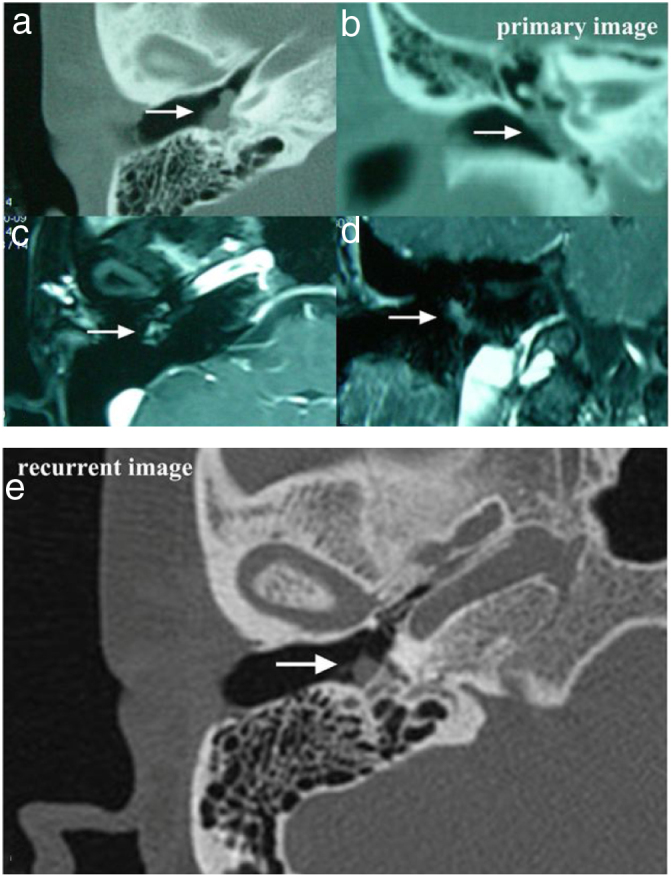
The temporal bone CT and MRI of Case 3. Images A, B, C, D showed the lesion was located in the meso- and hypotympanum with ossicular chain encasement on temporal bone CT (A, axial; D, coronal), and moderately enhance with gadolinium administration on MRI (C, axial; D, cronal). Image e showed tumor recurrence on high resolution temporal bone CT (E, axial), it demonstrated an isointensity mass located in the tympanum without bone erosion.

Case 2 and Case 3 presented with gray-white, non-pulsatile masses medial to a bulging tympanic membrane on otoscopy. The temporal bone Computed Tomography (CT) and Magnetic Resonance Images (MRI) showed the lesions in both cases were located in the meso- and hypotympanum with ossicle encasement (Case 3, Fig. 1A). However, both of the cases were intraoperatively confirmed that the lesion was located regionally in the meso- and hypotympanum, and the ossicles were intact without involvement by the tumor. Case 2 underwent CWU tympanomastoidectomy with PORP reconstruction and was confirmed as MEANT postoperatively, and the margins were shown to be negative. Case 3 underwent tympanotomy and tympanum lesion excision. Histopathology confirmed the diagnosis MEANT. However, Case 3 had a local recurrence at 38 months postoperative (Fig. 1E). The patient underwent a second surgery, CWU tympanomastoidectomy with intact ossicles, and then was followed up at 65 months with no evidence of recurrence.

**Figure 2:**
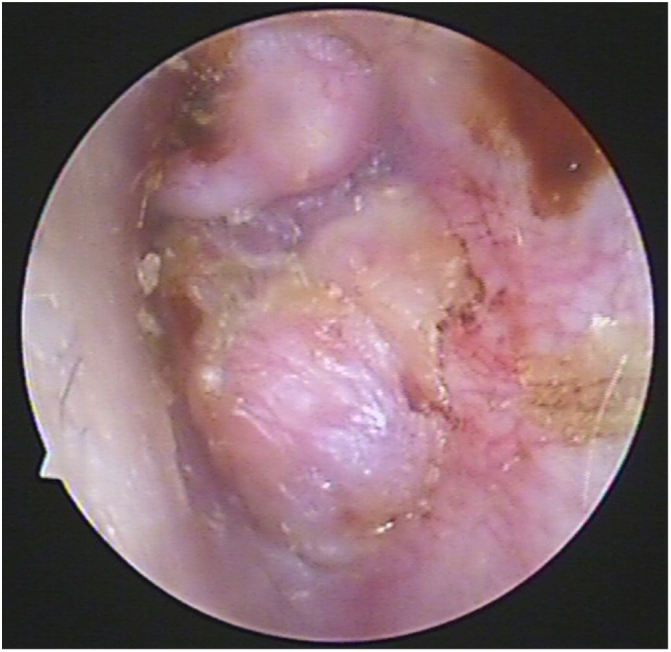
Otoscopical image of Case 6. Otoscopical image demonstrated a pink, hypervascular and non-pulsatile mass protruding into the external auditory canal.

**Figure 3:**
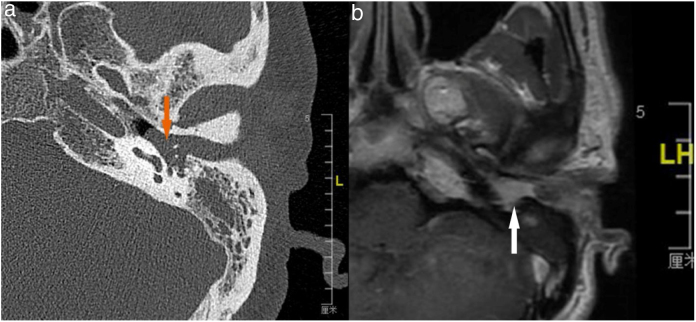
The temporal bone CT and MRI of Case 6. Temporal bone high resolution CT and temporal bone MRI with enhancement shows the lesion filled in the external auditory canal and tympanum with ossicular chain encasement and partially absorption (A, CT axial), and moderately enhanced with gadolinium administration on temporal bone MRI (B, MRI axial).

**Figure 4:**
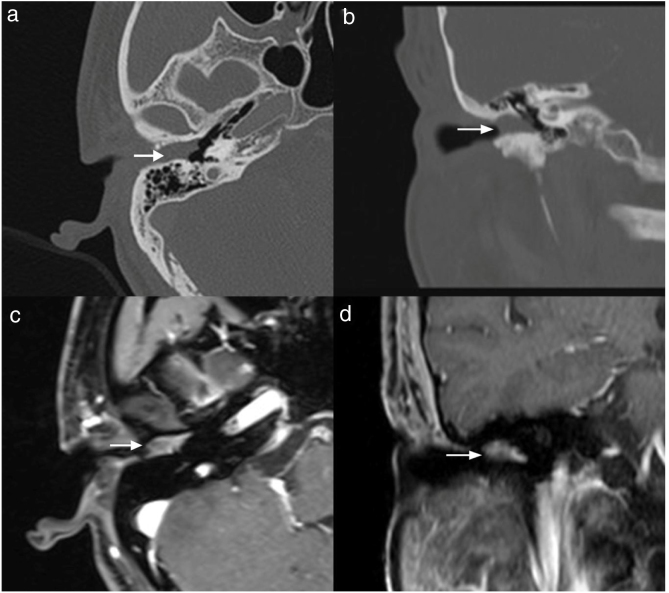
The temporal bone CT and MRI of Case 4. Temporal bone CT and MRI showed the lesion limited in EAC, without ossicular chain involved and bone erosion (A, axial; B, coronal). The mass was moderately enhanced with gadolinium administration on temporal bone MR (C, axial; B, coronal).

Case 4, 5 and 6 presented as a pink, hypervascular and non-pulsatile mass protruding into the External Auditory Canal (EAC) (Case 6, Fig. 2). Temporal bone CT and MRI with enhancement showed the lesions in each case involved the tympanum and/or EAC, with tumor encasement of the ossicles with partial absorption (Case 6, Fig. 3B and Case 4, Fig. 4C). LTBR was performed with EAC blind sac closure, packing the cavity with abdominal fat. Case 4 was combined with a superficial parotidectomy because of a histopathological diagnosis of an adenocystic carcinoma in a local hospital; however, the diagnosis was confirmed as MEANT postoperatively. The postoperative immunohistochemistry staining of all 6 patients is shown in Table 3. No patient underwent adjuvant therapy.

**Table 3:** Immunohischemistry staining of patients.

Case	CK	CK8	Syn	NSE	Vim	CD56	S-100	Ki-67	P63
1	++	/	+	−	+	/	/	−	/
2	+	/	+	+	+	−	−	−	+
3	+	+	+	+	+	−	+	/	/
4	+	+	+	+	+	/	−	≤20%	−
5	+	+	+	−	+	+	−	≤1%	+
6	+	/	+	/	+	+	−	≤1%	/

The cytokeratin cocktail and vimentin immunostaining; and neuroendocrine markers, such as Neuron-Specific Enolase (NSE), chromogranin, and uniform distribution of synaptophysin.

### Follow-up and outcomes

All patients were followed-up at 3 and 6 months postoperatively, and then every 6–12 months. The mean follow-up time was 80.7 months (range, 30–170 months). All patients, except Case 3 exhibited no evidence of recurrence during the period of follow-up.

## Discussion

Carcinoid/adenoma tumors have both epithelial and neuroendocrine components and are derived from primitive precursor or neural crest cells. The most common locations for such tumors are the gastrointestinal tract (73.7%) and the bronchopulmonary system (25.1%).^9^ Head and neck carcinoid/adenoma tumors are extremely rare and MEANTs are especially uncommon.

In our study, the median age at diagnosis was 43.5 (range, 30–61), and the male-to-female ratio was 1:1, which is consistent with the data reported in the literature. MEANTs tend to occur in the fifth decade (14–80 years) of life and affect both sexes equally.^4^ Symptoms at presentation are non-specific, including progressive conductive or mixed hearing loss (100%), a feeling of aural fullness (83.3%), tinnitus (66.7%), and otorrhea (33.3%), which frequently mimicks more common middle ear pathology such as temporal bone paraganglioma, CSOM, and cholesteatoma at initial clinical evaluation. No patients presented otalgia, bloody discharge, facial weakness or paralysis, vertigo, or carcinoid syndrome (Table 1). On presentation, otoscopy reveals a pink-hypervascular/gray-white, non-pulsatile mass medial to a bulging tympanic membrane or protruding into the EAC. There is no imaging study to definitively diagnose MEANTs; however, temporal bone CT and MRI with enhancement can be useful to evaluate the lesion's location, extension preoperatively, and assist scheduling management strategies.

Because of the non-specific presentation of MEANTs morphology under microscopic examination, its definitive pathological diagnosis is made by immunohistochemistry and ultrastructural examination of biopsy tissue. Immunohistochemical results can be useful parameters in differentiating MEANTs from other neuroendocrine tumors, such as paraganglioma. Typically, the tumor is Cytokeratins (CK), Vimentin (Vim) and chromogranin positive, with a lesser number of tumors proving to be Neuron-Specific-Enolase (NSE), Synaptophysin (Syn), serotonin and S-100 protein positive.^10^ In the present study, CK, Syn, and Vim were positive in all patients, and some patients also showed positive staining with CK8, NSE, CD56 and Ki-67 (≤1% or 20%). All patients presented negative for S-100 except for Case 3 (Table 3). However, these markers are not always sufficiently sensitive or specific to distinguish MEANTs. Recently, insulinoma-associated protein 1 has been reported as having excellent sensitivity and specificity, a standalone first-line marker of neuroendocrine differentiation for tumors of the head and neck.^11^ Nevertheless, the relationship between the immunohistochemistry staining results and the biological characteristics of MEANTs (such as recurrence, invasiveness and metastasis) remains elusive.

Although these tumors have long been considered to be indolent, of low aggressiveness, and benign neoplasms with rare metastases, the overall local recurrence rate was reported as 22%. These typically presented late, on average 11 years after treatment (range, 13 months to 33 years).^5^ Approximately 9% of patients even developed regional and distant metastasis, most commonly to the parotid lymph nodes, cervical lymph nodes, liver, and bone.^5^, ^12^, ^13^, ^14^, ^15^ Rare patients exhibited facial nerve paralysis, which may occur as a result of neural compression rather than perineural invasion.^6^ Local recurrence usually occurs after incomplete and conservative excisions, although some can be accepted as residual tumor.^5^ It is very important to schedule a suitable surgical approach tailored on the basis of the clinical and radiologic findings, and if necessary, procedures should be altered intraoperatively in order to completely remove the lesions with safe margins.

In our experience, complete surgical excision of the lesion is the primary curative treatment. In this study, 3 patients underwent CWU tympanomastoidectomy, including one patient with recurrence (Case 3) who underwent a tympanotomy and tympanum lesion excision in the initial management strategy, and another patient (Case 1) misdiagnosed and treated as CSOM. The other 3 patients underwent LTBR with the cavity being packed using abdominal fat. No adjuvant therapy was performed. All of these 6 patients showed a favorable prognosis without evidence of recurrence or metastases at the end time-point of follow-up.

There is a lack of universal consensus on the treatment strategies for MEANTs. A wide variety of surgical approaches have been used to deal with this disorder including excision of EAC mass, tympanotomy with transcanal/postauricluar approaches, radical mastoidectomy, canal wall-up/down tympanomastoidectomy, LTBR, Subtotal Temporal Bone Resection (STBR), and subtotal petrosectomy.^4^, ^5^, ^16^, ^17^ Ramsey et al.^5^ noted that tympanotomy was associated with a local recurrence rate of 29%, whereas radical mastoidectomy was associated with a local recurrence rate of 10%. Our experience suggested that local resection or tympanotomy is not recommended for managing MEANTs. It is difficult to be certain the lesions are removed completely, even with negative margins. It is especially challenging when the ossicles are partly encased. CWU tympanomastoidectomy or LTBR depends on the extent of the lesions, and potential adherence or invasiveness to critical structures, such as the petrous ICA and facial nerve.

As ossicular chain involvement is common, and management of the ossicular chain plays an important role in primary and recurrent disease. In the current study, the only one patient who developed recurrent disease had an intact ossicular chain retained at initial treatment. We suggest removing ossicular chain for MEANTs. Torske et al.^6^ concluded that the rate of recurrence increased in cases with ossicular chain involvement in which the ossicular chain is not removed. These findings were corroborated by Saliba and Evrard,^10^ who reported that in all recurrences the initial excision was conservative, leaving the ossicular chain intact at initial surgery. Moreover, for the patients with unresectable metastatic disease or aggressive cases, the treatment should involve a combination of surgical resection and comprehensive therapy, including postoperative adjuvant radiotherapy.^5^, ^15^, ^18^

## Conclusions

Patients with MEANTs usually present with non-specific clinical manifestations. Complete surgical resection is the primary curative treatment, and the ossicular chain should be removed. Surgical procedures depend upon the extent of the lesion. The propensity for local recurrence and invasiveness, as well as regional or distant metastasis of these tumors, make long-term follow-up and observation a necessity. Because of the extremely rare morbidity, each new case report and further multi-center investigations can help delineate the characteristics of the disease and thus establish a standard treatment protocol.

## Funding

This work was supported by grants from the 10.13039/501100001809National Natural Science Foundation of China (81660173), and the Science and Technology Program of Health and Family Planning Commission of Jiangxi Province (20175234).

## Conflicts of interest

The authors declare no conflicts of interest.
